# Deconstructing demographic bias in speech-based machine learning models for digital health

**DOI:** 10.3389/fdgth.2024.1351637

**Published:** 2024-07-25

**Authors:** Michael Yang, Abd-Allah El-Attar, Theodora Chaspari

**Affiliations:** ^1^ Computer Science & Engineering, Texas A&M University, College Station, TX, United States; ^2^ Computer Science & Engineering, Texas A&M University Qatar, Al Rayyan, Qatar; ^3^ Institute of Cognitive Science & Computer Science, University of Colorado Boulder, Boulder, CO, United States

**Keywords:** speech, machine learning, anxiety, depression, demographic bias, fairness

## Abstract

**Introduction:**

Machine learning (ML) algorithms have been heralded as promising solutions to the realization of assistive systems in digital healthcare, due to their ability to detect fine-grain patterns that are not easily perceived by humans. Yet, ML algorithms have also been critiqued for treating individuals differently based on their demography, thus propagating existing disparities. This paper explores gender and race bias in speech-based ML algorithms that detect behavioral and mental health outcomes.

**Methods:**

This paper examines potential sources of bias in the data used to train the ML, encompassing acoustic features extracted from speech signals and associated labels, as well as in the ML decisions. The paper further examines approaches to reduce existing bias via using the features that are the least informative of one’s demographic information as the ML input, and transforming the feature space in an adversarial manner to diminish the evidence of the demographic information while retaining information about the focal behavioral and mental health state.

**Results:**

Results are presented in two domains, the first pertaining to gender and race bias when estimating levels of anxiety, and the second pertaining to gender bias in detecting depression. Findings indicate the presence of statistically significant differences in both acoustic features and labels among demographic groups, as well as differential ML performance among groups. The statistically significant differences present in the label space are partially preserved in the ML decisions. Although variations in ML performance across demographic groups were noted, results are mixed regarding the models’ ability to accurately estimate healthcare outcomes for the sensitive groups.

**Discussion:**

These findings underscore the necessity for careful and thoughtful design in developing ML models that are capable of maintaining crucial aspects of the data and perform effectively across all populations in digital healthcare applications.

## 1 Introduction

In recent years, the field of digital healthcare has witnessed remarkable advancements, driven by the prolific collection of vast and diverse datasets and the application of cutting-edge machine learning (ML) algorithms (1). These advancements offer the promise of achieving improved healthcare outcomes through detailed data analysis and the generation of novel insights. However, within this landscape of opportunity lies a pressing concern related to the potential bias inherent in the data and the fairness of the algorithms employed. Data collected for digital healthcare applications often suffer from non-representativeness, which can lead to skewed and biased decision-making processes (2). Furthermore, machine learning algorithms, if not carefully designed and monitored, have the propensity to perpetuate existing biases present in the data, thereby exacerbating existing disparities in healthcare (3, 4). Given these inherent risks associated with ML in digital healthcare, recent administrative and regulatory efforts, including the European Union’s (EU) AI Act (AIA) (5) and the “Blueprint for an AI Bill of Rights” by the U.S. White House (6), have taken the initiative to lay out core principles that should guide the design, use, and deployment of AI to ensure an inclusive progress that does not come at the expense of traditionally underserved groups.

Speech-based ML technologies have observed an increased focus by digital healthcare due to the fact that speech can be unobtrusively collected via smartphones and wearable devices on a continuous basis, and carries valuable information about the human behavior and mental state. Speech is a result of the complex interplay between cognitive planning and articulation (7). The cognitive component of speech production involves cognitive planning via the formation of the message that a speaker intents to communicate. The motor component of speech production, also referred to as “articulation,” relies on the coordination of the lungs, glottis (i.e., including the vocal cords), and vocal tract (i.e., mouth, nasal cavity) (8). Both the motor and cognitive components of speech can be affected by the speaker’s traits and states, the first reflecting one’s permanent characteristics (e.g., race/ethnicity, gender) and the latter varying over time (e.g., emotion, stress, health condition) (9). Due to this richness of information, acoustic measures derived from speech, such as prosody or spectrotemporal characteristics, can reflect information that is critical for mental healthcare applications (e.g., stress, emotion, depression), while at the same time they can be confounded by demographic factors. The effect of state and trait characteristics on acoustic measures has been explored for each variable of interest separately. For example, previous studies support that acoustic measures vary between female and male speakers (10), and demonstrate the dependence of acoustic measures on race and ethnicity (11–13). At the same time, the effect of behavioral and mental health states on acoustic measures has been investigated in prior work in affective computing (14). Yet, limited work has examined whether ML systems for state recognition based on speech can yield differential results among demographic groups (15, 16).

Here, we examine demographic bias in speech-based ML systems that detect outcomes relevant to digital healthcare. We investigate potential differences in acoustic measures and labels between groups defined via gender, race/ethnicity, and their intersection. We further investigate the extent to which ML systems trained on these acoustic measures can preserve significant differences among groups in terms of labels (when applicable), and whether they depict differential performance among the considered groups. Finally, we study de-biasing methods that rely on removing features indicative of demography from the feature space, and transforming the feature space via adversarial learning to reduce evidence of the demographic information while preserving information about the focal behavioral and mental health state. We present our analysis via two case studies; Study 1 focuses on estimating anxiety levels and Study 2 on detecting depression from acoustic measures. Results indicate a significant dependence of the considered acoustic measures on gender and race/ethnicity. Despite the significant difference among groups in terms of the considered anxiety and depression labels, the ML systems were partially able to preserve those differences. Finally, we observe differences in ML performance among groups, which are partially mitigated via the de-biasing methods. Implications of these are discussed in the context of accelerating equitable ML decision-support algorithms for digital healthcare.

The contributions of this paper, in comparison to previous research, can be summarized as follows: (1) Prior studies (10–12) have explored demographic differences in acoustic features independently of ML algorithms. However, in Studies 1 and 2, we delve into the examination of acoustic feature differences among demographic groups as a potential source of algorithmic bias in speech-based ML decision-making; (2) Diverging from earlier research that primarily focused on disparities in ML performance among different groups (17–20), Studies 1 and 2 underscore the significance of preserving potentially meaningful distinctions among populations. Our findings reveal that, despite meaningful observed differences among groups in terms of anxiety and depression labels, these differences are not maintained in the ML decisions; and (3) There has been limited exploration of the effectiveness of de-biasing methods in the domain of behavior and mental health analytics (16). Hence, Study 2 contributes to expanding our understanding on how these methods perform when applied to speech analytics.

The remainder of this paper is structured as follows. Section 2. discusses the relationship between speech on demographic factors, the impact of anxiety and depression on acoustic features, and reviews prior work on algorithmic bias in healthcare and other high-risk applications. Section 3. presents Study 1 focusing on the examination of algorithmic bias when estimating anxiety levels using acoustic measures. Section 4. delves into Study 2, which explores the analysis and mitigation of algorithmic bias for depression detection. Section 5. provides an overview of the findings from both studies and discusses those findings in relation to prior work, delineating implications of these findings for fostering equitable digital healthcare. Finally, Section 6. summarizes the conclusions drawn from this work.

## 2 Prior work

### 2.1 The dependence of speech on demography

Differences in anatomical structure between male and female speakers, such as vocal fold size and vocal tract length, have been widely investigated (21) and serve as a main contributing factor to the observed significant differences between the two in terms of acoustic measures, such as fundamental frequency (F0) and formant frequencies (22). Beyond the differences in anatomy, behavioral factors might further result in acoustic differences between female and male speakers. For example, Sachs et al. found that in an effort to comfort with stereotypes, male speakers will sometimes speak with unnaturally low F0 and female speakers with an unnaturally high F0 (10). Other work has examined potential differences of acoustic measures based on race and ethnicity with somewhat conflicting results. Xue & Hao found differences in vocal tract diameters among White American, African American, and Chinese male and female speakers, which could serve as a factor of variation in acoustic measures (23). Lass et al. demonstrated that naive listeners can distinguish between 10 African American and 10 White speakers, balanced in terms of gender, with approximately 75% accuracy when listening to full sentences (24). Perceptual differences in speech between African American and White speakers have been observed (25), a finding with important social implications such as racial or ethnic profiling (26). In terms of acoustic analysis, Sapienza analyzed F0 values of 20 African American and 20 White adult speakers (balanced for gender) and did not find any significant differences between the two (13). Li et al. demonstrated no difference between African American and White men in F0 mean and range, but African American women produced consistently lower mean F0 than White women (12). Hispanic female speakers further showed the highest values of jitter in a sustained vowel task (27). Cantor-Cutiva et al. examined differences in vocal measures between English speakers and bilingual English-Spanish speakers (11). Results indicate that monolingual speakers depicted higher F0 mode compared to bilingual English-Spanish speakers. Bilingual male speakers had higher jitter than monolingual speakers, while bilingual female speakers had lower jitter and shimmer than monolingual speakers.

### 2.2 The impact of anxiety on speech

Evidence from prior work indicates that the sympathetic activation caused by high state anxiety can produce an increase in lung pressure, subglottal pressure, irregular palpitation of the vocal folds, and vocal tremor, that can cause voicing irregularities and discontinuities in frequency contours (28). These can be quantified via changes in acoustic measures, such as F0, jitter, shimmer, and vocal intensity (29, 30). For instance, Van Lierde et al. examined the voice of female speakers in a stress-inducing task (i.e., reading a passage before an audience of 70 people) and found that it was more breathy and strained (31). Özeven et al. found an increase in F0 and marginal increase in jitter when participants suffering from social anxiety were asked to read the Beck Anxiety Inventory (32). In a similar context, Weeks et al. demonstrated that male patients suffering from social anxiety disorder depict increased F0 during a speaking task compared to participants of the non-socially anxious control group (30). Prior work indicates that jitter and shimmer are associated with increased emotional arousal (33, 34). Review studies further support an increase in F0 during stressful tasks, but these trends are not universal (29). For example, Van Lierde et al. observed lower objective vocal quality in female speakers during the stress inducing task, characterized by lower F0, lower frequency, and intensity. Kappen et al. examined the associations between negative affect elicited from a stress inducing task and acoustic measures of F0, ratio of the energy of the first formant (F1) to the energy of the second formant (F2), and harmonics-to-noise ratio (HNR) (35). Results via a network analysis indicated that jitter was the only speech parameter that was directly connected to change in negative affect with a positive association between the two. Finally, Jiang et al. demonstrated that speakers perceived to be confident speak with higher voice intensity compared to the non-confident ones (36).

### 2.3 The impact of depression on speech

Mental health disorders, such as depression, can produce noticeable changes in speech patterns and yield prosodic abnormalities (see (37) for a review). Findings from prior work suggest that patients with depression are characterized by decreased speech loudness, slowed speech rate, and monotonous pitch (38). Prosodic timing measures, such as pause time, speech pause ratio, and speaking rate have been found important indicators of depression (39). Glottal measures that capture the association between volume and velocity in the airflow have been further successfully used for classifying between healthy participants and participants with depression (40). Other work has further examined features that reflect changes in the coordination of the vocal tract motion across different time scales and formant frequencies (41). Beyond feature analysis, prior studies have combined the prosody, source features, formants, and spectral features with ML models for automatically identifying depression (40, 42–44).

### 2.4 Bias in human-centered machine learning

Recently there has been an upsurge of attention on identifying and correcting algorithmic bias. Early efforts focused on generic tasks of image processing (45, 46), natural language processing (47), and speech recognition (48, 49). Emerging work has started to discuss algorithmic bias in specific domains, such as healthcare (50).

In healthcare, Obermeyer et al. investigated sources of racial bias in commercially available ML algorithms that are used to recommend preventive care to patients based on their health biomarkers (e.g., cholesterol, hypertension, diabetes severity) (17). Results indicate that when these algorithms were trained based on healthcare costs as an outcome, they predicted significantly higher risk scores for White patients compared to African American patients, thus rendering White patients significantly higher chance of getting recommended for preventive care. This bias was mitigated when the number of active chronic conditions was used as the outcome. Raza predicted re-hospitalization likelihood of patients with diabetes based on their medical records, medication, and biomarkers (19) and used various de-biasing techniques, such as re-weighting the samples from the sensitive groups during training (51), applying adversarial learning for reducing the evidence of sensitive attributes in the data (52), and transforming features to improve group fairness, as well as both individual and group fairness (53). Results indicate that sample re-weighting yields classification accuracy similar to the original ML model, while also improving upon the disparate impact metric compared to all the considered algorithms (53). Park et al. used ML to predict postpartum depression (PDD) based on demographics, pregnancy outcomes, psychiatric comorbidities, medication use, and healthcare utilization (18). Results on approximately 300,000 White and 200,000 African American participants with matching age and insurance enrollment indicated that the first group was twice as likely to be evaluated for and diagnosed with PPD. The authors attempted to remove bias via re-weighting the samples between the two groups and regularizing the dependency between race and PDD outcome during training. Both methods yielded improved disparate impact compared to the original ML algorithm, even when removing race from the input. The re-weighting method further yielded equivalent positive PDD rates between groups. Zanna et al. explored demographic bias in anxiety prediction using 10-week long electrocardiogram (ECG) data from 200 hospital workers (20). Algorithmic bias was found in terms of age, income, ethnicity, and whether a participant was born in the U.S. or not. The authors further proposed a multitask learning approach to predict anxiety and one of the demographic labels. They introduced a Bayesian approach that chose the trained model whose weights depicted the highest uncertainty about the sensitive demographic label, thus, yielding low predictive power of that outcome and reduced demographic bias.

## 3 Study 1: bias related to gender and race/ethnicity when estimating anxiety levels from speech

The goal of this case study is to identify demographic bias in terms of gender, race/ethnicity, and their intersection when using acoustic measures to estimate anxiety. We explore different sources of bias that can potentially be present in different stages from the data origins to the model outcomes. In order to reduce the complexity in the learning stage, we explore a simple ML model, namely a linear regression model that estimates public speaking outcomes based on acoustic measures, which allowed us to constrain the effect of confounding factors related to ML training (e.g., hyper-parameter tuning).

### 3.1 Data description

The data for this case study come from the VerBIo dataset, a multimodal bio-behavioral dataset of individuals’ affective and stressor responses in real-life and virtual public speaking settings (54). The VerBIO dataset contains data from both Native and non-Native English speakers. Due to the inherent differences in accent between the two groups that can bias the acoustic descriptors and introduce significant confounding factors in the analysis, the data for this case study include the 30 Native English speakers (14 female, 16 male). The decomposition of the considered participants in terms of gender, race, and ethnicity is shown in Table 1. While the distribution of participants is well-balanced in terms of gender, our data has a larger number of White Americans compared to African Americans and participants of Hispanic origin. Since each speaker conducted more than one public speaking sessions, the total number of audio files per group is reported in Table 2.

**Table 1:** Number of speakers per demographic group.

	African American	Hispanic	White American	Total
Female	3	2	9	14
Male	3	7	6	16
Total	6	9	15	30

**Table 2:** Number of audio samples per demographic group.

	African American	Hispanic	White American	Total
Female	21	11	75	107
Male	30	47	45	122
Total	51	58	120	229

### 3.2 Methods

The considered outcome in our analysis is the self-reported anxiety that was captured via the State-Anxiety and Enthusiasm (SAE) scale. The input features of the model include the F0 on a semitone frequency scale, loudness, jitter, and shimmer, since these are commonly used indicators of state anxiety and fear of the public speaking encounter (54–56). Choosing a small number of indicative and interpretable features allows us to focus the analysis on the factors that have the most significant impact on the problem at hand, in this case, the effect of anxiety on acoustic features. Voice activity detection was conducted before extracting the acoustic measures. Acoustic measures were extracted using the openSMILE toolkit within each utterance. The average measure over all utterances of an audio file was subsequently considered in the analysis.

First, we explore bias in the input data. We report the mean and standard deviation of the acoustic features and the anxiety outcome per demographic group. Due to the physiological differences between female and male voices (57), the two groups are considered separately within each race/ethnicity when examining the acoustic features. In order to determine the extent to which the considered groups are different in terms of input and output data, we conduct a two-way analysis of variance (ANOVA) with gender and race/ethnicity as independent variables. The dependent variables in the ANOVA include the acoustic measures and the self-reported anxiety outcome. Following that, we conduct a post-hoc analysis with t-tests to identify significant differences between specific pairs of groups.

Second, we explore bias in the outcome of the ML model. We train a linear regression model that estimates the anxiety outcome based on the four acoustic measures. We opted for this shallow model because of its low data requirements and since similar models are widely used in healthcare and edge computing applications (18, 19). We obtained similar results using decision tree regression and random forest regression, thus, we will focus on the linear regression in the rest of the paper. A leave-one-speaker-out cross-validation is conducted, according to which samples from each speaker serve as the test data in each fold and the rest of the speakers are included in the training data. This process is repeated as many times as the total number of speakers. The estimated anxiety outcome from each fold is collected and used in the subsequent analysis. We report the mean and standard deviation of the anxiety outcome that was estimated by the linear regression model. We further conduct a similar statistical analysis that includes a two-way ANOVA and post-hoc tests, with the anxiety outcome serving as the dependent variables, and gender and race/ethnicity as the independent variables. We computed the absolute relative error (RE) between self-reported anxiety and anxiety estimated by the ML model, as a measure of the overall performance of the ML system. In addition, we computed the equality of opportunity (EO) between each of the sensitive and the corresponding non-sensitive groups as EO=1−‖RE(sensitive)−RE(non−sensitive)‖. Values of EO close to 1 indicate that outcomes for the sensitive and non-sensitive groups are estimated with similar accuracy. When examining the intersection between race and gender, we considered the White female or White male speakers as the non-sensitive group and the other groups as the sensitive group. When looking at gender alone, we considered the male speakers as the non-sensitive group and compared them with the female speakers. When looking at race along, we considered the White speakers as the non-sensitive group and the African American and Hispanic speakers as the sensitive group.

### 3.3 Results

The statistics of each feature per demographic group are provided in Table 3, and the statistics of the actual and estimated anxiety per demographic group are in Table 4. Results from the ANOVA and post-hoc analysis are presented in Tables 5, 6, respectively.

**Table 3:** Mean and standard deviation of acoustic features per demographic group.

Group	F0	Loudness	Jitter	Shimmer
African American female	34.968 ± 1.636	0.645 ± 0.293	1.094 ± 0.137	0.025 ± 0.005
Hispanic female	35.800 ± 1.307	0.335 ± 0.066	1.104 ± 0.106	0.025 ± 0.004
White female	36.603 ± 1.837	0.766 ± 0.283	1.080 ± 0.115	0.022 ± 0.004
African American male	23.776 ± 2.339	0.689 ± 0.289	1.317 ± 0.141	0.024 ± 0.004
Hispanic male	26.771 ± 3.217	0.689 ± 0.240	1.242 ± 0.138	0.022 ± 0.005
White male	26.188 ± 1.757	0.726 ± 0.248	1.178 ± 0.128	0.020 ± 0.002

**Table 4:** Mean and standard deviation of anxiety labels and predictions per demographic group.

Group	Actual	Estimated
African American female	41.62 ± 6.95	50.06 ± 2.66
Hispanic female	54.73 ± 6.54	51.78 ± 1.98
White female	49.75 ± 9.98	49.96 ± 2.56
African American male	44.33 ± 9.28	51.25 ± 2.82
Hispanic male	56.77 ± 7.79	51.77 ± 2.19
White male	51.84 ± 6.39	51.89 ± 2.69

**Table 5:** 2 × 3 Analysis of variance (ANOVA) analysis examining significant differences among demographic groups.

Measure	Race/ethnicity	Gender	Interaction
F0	F(2, 223) = 19.96, p<0.01	F(1, 223) = 1083.710, p<0.01	F(2, 223) = 2.496, p<0.01
Loudness	F(2, 223) = 6.055, p<0.01	F(1, 223) = 1.948, p=0.16	F(2, 223) = 7.592, p<0.01
Shimmer	F(2, 223) = 15.024, p<0.01	F(1, 223) = 5.868, p<0.01	F(2, 223) = 0.523, p<0.01
Jitter	F(2.0, 223) = 6.800, p<0.01	F(1.0, 223) = 56.210, p<0.01	F(2, 223) = 4.120, p<0.01
Self-reported anxiety	F(2, 223) = 30.673, p<0.01	F(1.0, 223) = 3.489, p<0.01	F(2, 223) = 0.026, p<0.01
Estimated anxiety	F(2, 223) = 1.076, p=0.34	F(1.0, 223) = 14.928, p<0.01	F(2, 223) = 2.040, p=0.13

**Table 6:** Post-Hoc analysis examining significant pairwise differences between demographic groups.

Measure	AF-WF	HF-WF	AM-WM	HM-WM
F0	t(94) = 3.69, p=0.00	t(84) = 1.40, p=0.17	t(73) = 5.09, p=0.00	t(90) = −1.07, p=0.29
Loudness	t(94) = 1.72, p=0.09	t(84) = 5.00, p=0.00	t(73) = 0.58, p=0.56	t(90) = 0.72, p=0.47
Jitter	t(94) = −0.46, p=0.64	t(84) = −0.65, p=0.52	t(73) = −4.43, p=0.00	t(90) = −2.28, p=0.02
Shimmer	t(94) = −3.25, p=0.00	t(84) = −2.41, p=0.02	t(73) = −5.17, p=0.00	t(90) = −2.25, p=0.03
Self-reported anxiety	t(94) = 3.50, p=0.00	t(84) = −1.60, p=0.11	t(73) = 4.16, p=0.00	t(90) = −3.30, p=0.00
Estimated anxiety	t(94) = −0.15, p=0.88	t(84) = −2.26, p=0.03	t(73) = 0.99, p=0.32	t(90)=0.24, p=0.81

AF, African American female; HF, Hispanic female; WF, White female; AM, African American male; HM, Hispanic male; WM, White male.

Results indicate significant differences with respect to F0, jitter, and shimmer for gender, race/ethnicity, and their interaction (Tables 5). Based on the post-hoc analysis (Table 6), loudness depicts significant differences for race/ethnicity, but not for gender. White female speakers depicted significantly higher F0 compared to African American female speakers. Similarly, White male speakers depicted a significantly higher F0 compared to African American speakers. The loudness exhibited by white female speakers was significantly higher than that observed in Hispanic female speakers. Moreover, white female speakers also demonstrated higher loudness levels in comparison to African American female speakers, although this difference was not statistically significant (p=0.09). Hispanic and African American female speakers displayed significantly higher values of jitter compared to White female speakers. Hispanic and African American male speakers exhibited significantly higher values of jitter and shimmer compared to White male speakers.

The ANOVA revealed the presence of significant differences concerning gender, race/ethnicity, and their interaction in relation to self-reported anxiety (Table 5). Subsequent post-hoc analyses (Table 6) indicated that Hispanic female speakers reported the highest levels of anxiety, although the difference in this measure between Hispanic female speakers and White female speakers was not statistically significant (p=0.11). Conversely, African American female speakers reported the lowest levels of anxiety, a difference that achieved statistical significance when contrasted with White female speakers. Additionally, African American male speakers reported significantly lower anxiety levels compared to their White male counterparts. In parallel, Hispanic male speakers reported the highest levels of anxiety, a difference that was statistically significant when compared to White male speakers.

Results finally indicate that the anxiety estimates provided by the ML model do not necessarily follow the patterns of the actual values (Table 4). For example, Hispanic speakers depicted the highest levels of state anxiety, but this ordering is not reflected in the ML estimates (Table 4). The significant differences among groups with respect to self-reports are further not maintained in the estimated anxiety values (Tables 5, 6). This might suggest that the ML system is not able to maintain potentially meaningful differences between demographic groups. Regarding variations in ML performance across demographic groups (Table 7), the ML system exhibits the greatest level of error, RE, in anxiety estimation when analyzing African American female speakers, African American male speakers, and their combination, in comparison to their respective demographic counterparts. Consequently, the EO is lower for the African American group, indicating the ML system does not estimate the anxiety labels equally well for the African American group compared to the White American group. When segregating by gender alone, the ML system yields higher RE in anxiety estimation and lower EO for female speakers when contrasted with male speakers.

**Table 7:** Absolute relative error (RE) and equality of opportunity (EO) per group.

Groups	RE	EO
AA females	0.093	0.984
Hispanic females	0.055	0.977
White females	0.077	–
AA males	0.089	0.956
Hispanic males	0.078	0.967
White males	0.045	–
African American	0.090	0.975
Hispanic	0.074	0.991
White	0.065	–
Females	0.075	0.976
Males	0.051	–

## 4 Case study 2: gender bias in detecting depression

The goal of Case Study 2 is to explore gender bias in a speech-based ML algorithm used to detect depression. We explore potential gender bias using a set of acoustic features that represent time-based, frequency-based, and spectral balance parameters of speech that are commonly used for mental health tasks. We further investigate the effect of feature selection and transformation algorithms in reducing evidence of bias in the decisions of the ML models and their performance based on the aforementioned features. Finally, we investigate differences between depression labels and depression outputs estimated by the ML models for female and male speakers, in an attempt to understand to what extent the estimated rates of depression are different compared to the actual rates of depression for each group.

### 4.1 Data description

The data for this case study came from the Distress Analysis Interview Corpus Wizard of Oz (DAIC-WoZ) dataset, which consists of audio interviews from 107 participants (63 males and 44 females) (58). A participant was assigned to the depression class if their score in the Patient Health Questionnaire (PHQ-8) was greater than 9; otherwise they were assigned to the healthy class. Thirty one participants were classified as having depression (14 males and 17 females), and the remaining as healthy. Each audio was split into individual utterances based on the transcripts, resulting in 16,906 utterances from all participants.

### 4.2 Methods

Acoustic measures were extracted at the utterance-level and included the 88 features of the extended Geneva Minimalistic Acoustic Parameter Set (eGeMAPS) that include parameters related to frequency, energy/amplitude, spectral balance, and timing (59). Our analysis focused on classifying between depression and no depression. Due to the binary nature of this classification task, prior work on the same task has achieved good performance with accuracies of around 70%–80% (60). In contrast, the automatic estimation of the degree of depression severity is a more challenging task with prior work indicating a root mean square error of 5–7 and the PHQ-8 scale ranging between 0–27 (61). Considering that the study of algorithmic bias for automatically detecting depression from speech is at its infancy, we decided to focus on the binary classification task.

We implemented two debiasing methods. The first is a feature selection method in which we remove from the input of the ML model the features that depict the highest discriminative ability of gender. We conducted a t-test analysis for each of the aforementioned features and measured the extent to which they are significantly different between female and male speakers. We ranked those features in increasing order of p-value and removed the M features with the lowest p-value, where M=5,10,15,20,30,60, to include as an input in the ML model. The second de-biasing method is a feature transformation approach that modifies the original input space so that it becomes less predictive of gender, while still preserving its discriminative ability of depression. Grounded in prior work on adversarial learning that depicts promising results for this purpose (62, 63), we leverage an auto-encoder architecture that takes as an input the original feature vector and yields an output feature vector via a non-linear transformation implemented through an autoencoder. The autoencoder conducts an identity mapping to minimize the difference between the input and the output. At the same time, the bottleneck layer goes through two additional transformations. The first one outputs the gender of the speaker and the second one yields the depression outcome. The autoencoder is trained to minimize the cross-entropy function that corresponds to the depression outcome and maximize the cross-entropy function that corresponds to the gender. It comprises of 2 hidden layers, with 256 and 128 nodes in the encoding and decoding layers, respectively. We used the Rectified Linear Unit (ReLU) activation function and the Adam optimizer with a learning rate of 0.0001 to train the autoencoder for 50 epochs. Mean squared error served as the loss function.

In order to further improve depression classification performance, the Pearson’s correlation between the PHQ scores and the aforementioned features (i.e., the original features and the features resulting from the adversarial learning debiasing method) was computed. The top K features, where K=5,10,15,20,30,60,88, with the highest correlation were further selected to serve as an input to the ML model that conducted depression classification. The ML model comprised of a feedforward neural network that had 2 hidden layers with 32 nodes each and ReLU activation function, and an output layer with 2 nodes that corresponded to the depression classification outcome with softmax activation. Binary cross entropy was used as the optimization loss. We trained the network using the Adam optimizer with a learning rate of 0.001 for 100 epochs and a mini batch size of 32. Early stopping was incorporated to optimize training performance. The final depression decision was taken at the participant-level by aggregating the individual decisions of the network from all utterances and taking their maximum. A leave-one-participant-fold-out cross-validation was conducted, in which samples from each participant fold (i.e., each fold consisted of samples from 10 participants) serve as the test data and the rest of the folds are included in the training data. This process was repeated as many times as the total number of participant folds.

Evaluation of the results was conducted via three metrics: (1) balanced accuracy (BA) that computes the average of true positive rate (TPR), corresponding to the depression class, and true negative rate (TNR), corresponding to the healthy class; (2) equality of opportunity (EO) that computes the difference in TPR between female and male participants, i.e., EO=1−‖TPR(male)−TPR(female)‖, quantifying to what extent the same proportion of female and male participants receive a true positive outcome (53); and (3) predicted positive rate (PPR) for female speakers, computed as the percentage of female speakers from the whole population of female speakers that were assigned to the depression class by the ML, and the PPR for male speakers, computed as the percentage of male speakers from the whole population of male speakers that were assigned to the depression class by the ML. The PPR for female and male speakers contributes to better understanding potential differences in which the two groups receive a positive ML outcome. The PPR for each group of speakers based on the ML estimations was compared with the corresponding base PPR based on the data labels using a two-proportion Z-test. Significant results from this test would indicate that the PPR derived from the ML estimations is significantly different from the base PPR obtained using the data labels.

### 4.3 Results

Here we present the results from Case Study 2. First, we discuss differences in labels between female and male speakers and the results of the machine learning models using the original acoustic features (Section 4.3.1). Following that, we describe the acoustic features that are the most indicative of depression, as well as the acoustic features that the most indicative of gender, and their potential overlap. We further discuss these in association to the results of the machine learning models that are trained using the features resulting from feature selection (i.e., selecting the most relevant features to depression, removing the most relevant features to gender) (Section 4.3.3). Finally, we present the results of the machine learning models yielding from further transforming the feature space with adversarial learning (Section 4.3.3). Evaluation metrics include the depression classification performance via BA and fairness metrics via EO and PPR.

#### 4.3.1 Results using the original features

The average PHQ-8 score for female and male speakers were 7.43 (±6.12) and 5.59 (±4.72), respectively. The t-test results between the two groups indicate evidence against the null hypothesis (t(105)=−1.723, p=0.087) without reaching statistical significance. When binarizing the PHQ scores, the depression base rates among female speakers in the dataset under consideration is 38.6% (i.e., 17 out of 44), while among male speakers, it stands at 22.2% (i.e., 14 out of 63). Similarly to the continuous PHQ-8 scores, the difference in actual depression rates between the two groups indicates evidence against the null hypothesis (z=−1.842, p=0.066), but without depicting statistical significance. This finding potentially aligns with empirical evidence suggesting higher depression rates among women, possibly attributed to psychological or biological factors (64–66).

**Figure 1:**
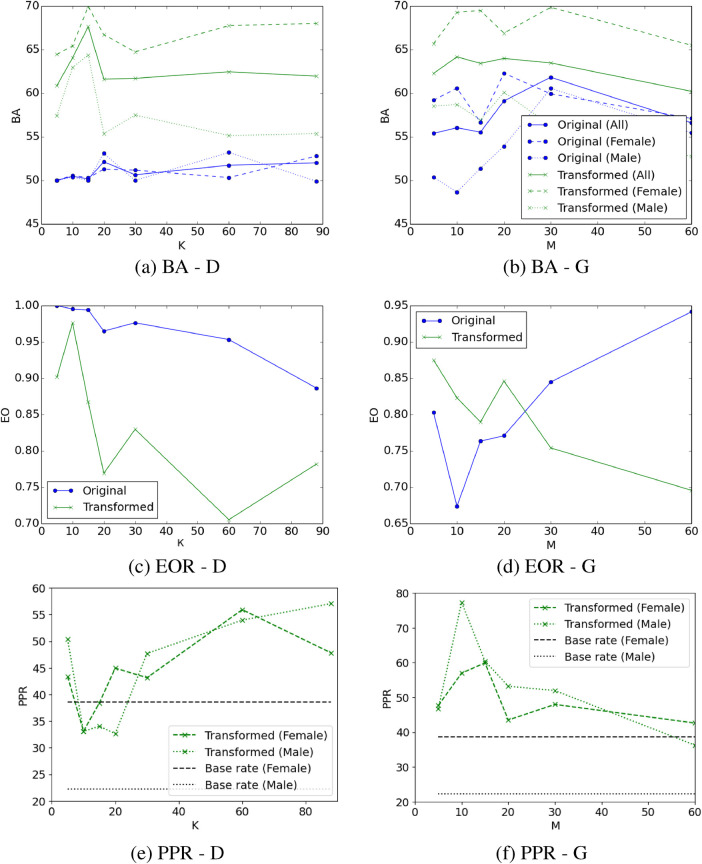
Balanced accuracy (BA), equality of opportunity (EO), and predictive positive rate (PPR) of depression when using the K acoustic measures most relevant to depression (D) (sub-figures **A**, **C**, **E**; blue lines), or after removing the M acoustic measures most relevant to gender (G) (sub-figures **B**, **D**, **F**; blue lines), and their transformation via adversarial learning (all sub-figures green lines). Same legend applies to subfigures (**A**) and (**B**). Feature transformation via adversarial learning improves performance. The optimal tradeoff between balanced accuracy and EO yields from the 10 most relevant features to depression after applying the adversarial learning transformation (sub-figures **A**, **B**; green line). All model configurations overestimate depression outcomes for male speakers (sub-figures **E**, **F**; green/black dotted lines), a finding which is statistically significant. The majority of model configurations overestimated depression for female participants, but also underestimated depression in three cases (sub-figures **E**, **F**; green/black dashed lines) with these results not being statistically significant. The difference between PPR and depression base rate in male speakers is higher compared to female speakers.

The original acoustic features depict BA of 52.79% for female speakers and BA of 49.90% for male speakers (Figure 1A, blue lines, last point corresponding to K=88). While these two BA metrics do not depict a statistically significant difference between the two groups (z=0.961, p=0.337), they are both close to chance accuracy indicating that the original set of features might not be effective in the considered depression classification system. Finally, based on the original features, the ML systems tend to underestimate depression for both female and male speakers (Table 8) depicting very low rates of depression estimation (i.e., 1 out of 44 for female speakers, 1 out of 63 for male speakers). These predicted depression rates are statistically different compared to the actual base depression rates for each group (z=−4.228, p=0 for female speakers; z=−3.969, p=0 for male speakers).

**Table 8:** Z-test results comparing the true depression rate and the estimated depression rate by machine learning based on the original features, the K transformed features most relevant to depression, and the M transformed features least relevant to gender for female and male speakers separately.

Outcome	Female speakers	Male speakers
Original features	z=−4.228,p=0.000	z=−3.576,p=0.000
Most relevant to depression (K=5)	z=0.434,p=0.665	z=3.331,p=0.001
Most relevant to depression (K=10)	z=−0.669,p=0.503	z=1.204,p=0.229
Most relevant to depression (K=15)	z=−0.220,p=0.826	z=1.392,p=0.164
Most relevant to depression (K=20)	z=0.648,p=0.517	z=1.204,p=0.229
Most relevant to depression (K=30)	z=0.434,p=0.665	z=2.990,p=0.003
Most relevant to depression (K=60)	z=1.496,p=0.135	z=3.669,p=0
Most relevant to depression (K=88)	z=0.861,p=0.389	z=4.006,p=0
Least relevant to gender (M=5)	z=0.861,p=0.389	z=2.818,p=0.005
Least relevant to gender (M=10)	z=1.707,p=0.088	z=6.236,p=0
Least relevant to gender (M=15)	z=1.919,p=0.055	z=4.174,p=0
Least relevant to gender (M=20)	z=0.434,p=0.665	z=3.500,p=0
Least relevant to gender (M=30)	z=0.861,p=0.389	z=3.331,p=0.001
Least relevant to gender (M=60)	z=−0.220,p=0.826	z=2.472,p=0.013

#### 4.3.2 Results using the selected features

Out of the twenty most discriminative features of depression, we got 6 frequency-based, 9 spectral, 4 energy-based, and 1 temporal (see Supplementary Table S1 for a detailed list). In addition, 11 of these features were also significantly different between female and male speakers. It is worth noting that the five most discriminative feature of depression, including the bandwidth of the third formant (“F3bandwidth_sma3nz_amean”), as well as the mean and $20^{\mathrm{th}}/50^{\mathrm{th}}/80^{\mathrm{th}}$ percentile of the semitone $F_{0}$ frequency (“F0semitoneFrom27.5Hz_sma3nz_amean,” “F0semitoneFrom27.5Hz_ sma3nz_percentile20.0,” “F0semitoneFrom27.5Hz_sma3nz_percenti le50.0,” “F0semitoneFrom27.5Hz_sma3nz_percentile80.0”) are also included in the list of the 20 most discriminative features of gender (see Supplementary Table S2). These indicate the dependency of features used to classify depression on gender.

When using the K features most relevant to depression as an input to the ML model, BA tends to increase above 50% and depicts differences between male and female speakers (Figure 1A, blue lines). Specifically, BA is higher for male speakers when choosing the K=20,60 features most indicative of depression, suggesting the presence of algorithmic bias against female participants. On the contrary, when using the K=30,88 features most indicative of depression, BA is higher for female speakers compared to male speakers, suggesting the presence of bias on the opposite direction (i.e., against male participants). However, we observe that EO measures tend to decrease below 1 as more acoustic features are incorporated into the model (Figure 1C, blue line). As we increase the number K of features that are the most relevant to depression, there is a risk of adding features to the model that could be influenced by gender. This might introduce bias in the decision of the model, which is evidenced by differential BA measures between male and female speakers, as well as EO measures falling below 1.

Training the depression classification models after removing the M features that are the most relevant to gender from the feature space overall improves the BA (Figure 1B, blue lines), surpassing the BA achieved when selecting the K features that are most relevant to depression (Figure 1A, blue lines). In this case, BA measures tend to be higher for female participants compared to male participants, indicating that this technique can potentially introduce bias in favor of female participants and against male participants, as also demonstrated by the EO measures which tend to be lower than 1 in the majority of cases (Figure 1D, blue line).

Predicted depression rates for both feature selection strategies are very low for both female and male speakers (i.e., ranging between 0%–6.6% for female speakers and 0%–0.76% for male speakers) and significantly different from the actual depression base rates. Consequently, for the sake of clarity in visualization, these rates are not depicted in Figures 1A, F. These suggest that feature selection alone cannot preserve the base depression rates exhibited in the original data.

#### 4.3.3 Results using the transformed features

When the adversarial learning transformation is applied to the K selected features, we observe a significant boost in BA. In this case, BA is consistently higher for female speakers compared to male speakers (Figure 1A, green lines). This suggests that de-biasing via applying adversarial learning to the K most relevant features to depression favors female participants who depict higher BA for depression classification compared to male participants. While the EO measure is much lower than 1 in many cases, there seems to be an optimal tradeoff between balanced accuracy and EO which occurs when we choose the K=10 most relevant features to depression followed by applying the adversarial learning transformation that reduces the evidence of gender in the corresponding measures (Figures 1A, C). In this case, BA is at similar levels for both genders (i.e., 65.44% and 62.96% for female and male speakers, respectively) and the EO is close to 1 (i.e., EO=0.976), suggesting that the selection of the most relevant features to the focal outcome followed by feature transformation via adversarial learning can reduce algorithmic bias.

Adversarial learning on the feature space that does not contain the M features most relevant to gender also improves the BA compared to using the same feature space without adversarial learning (Figure 1B, green lines). The optimal tradeoff between BA and EO metrics appears to be when we remove the M=5 features that are most relevant to gender followed by feature transformation with adversarial learning, yielding 62.28% BA (i.e., 65.7% for female speakers, 58.52% for male speakers) and 0.875 EO. However, these results are inferior compared to the optimal tradeoff achieved by keeping the K features that are most relevant to depression followed by feature transformation with adversarial learning (i.e., 65.44% BA for female speakers, 62.96% BA for male speakers, 0.976 EO). For the majority of cases, applying adversarial transformation to the feature space that does not contain the M features that are most relevant to gender seems to be less effective for mitigating bias yielding lower EO metrics, compared to applying the same transformation to the feature space with the K most relevant features to depression (Figures 1B, D, green lines).

The machine learning models that use the transformed features exhibit a tendency to overestimate depression among male speakers, as indicated by a higher estimated depression rate compared to the actual depression rate (see Table 8). The difference between actual and estimated depression rates for male speakers is statistically significant across all cases when utilizing the least relevant features to gender (Table 8, Figure 1E, green line), and in four out of the seven cases when employing the most relevant features to depression (Table 8, Figure 1F, green line). The ML models tend to underestimate the depression for female speakers when using the 10 and 15 most relevant features to depression or removing the 60 least relevant features to gender (Figures 1E, F). For the remaining of the feature transformation cases, the depression for female speakers is overestimated. These differences in the female speaker group are not statistically significant (Table 8).

## 5 Discussion

Significant differences among demographic groups based on commonly used acoustic measures were found in both Case Study 1 (Table 5), Table 6 and Case Study 2 (Supplementary Table S2), a finding which corroborates with prior work. In Study 1, for example, White female speakers depicted the highest F0 measures as in (10, 12), African American women had the lowest F0 similar to (12), and Hispanic female speakers depicted the highest jitter as in (11, 27) (Table 6). We also found significant differences in F0, jitter, and shimmer between African American and White male speakers (Table 6). These differences might be a result of anatomical factors (21, 23). Yet, part of these differences might be also attributed to the corresponding state of the speaker. For example, Hispanic speakers reported increased state anxiety during the public speaking presentation. This finding might further explain the high jitter values in this group that reflect increased speech trembling. Similarly, the larger set of energy, frequency, and spectral features in Study 2 depicted dependencies on both gender and depression (Supplementary Tables S1, S2). In Study 2, differences in between female and male speakers were observed in terms of various features that are discriminative of depression, and particularly frequency-based features (Supplementary Table S2). However, those differences were not statistically significant. This finding indicates that acoustic information related mental health is confounded by the speaker’s gender. This can potentially lead to unintended consequences when utilizing these features for automated depression detection, as a model that leverages frequency-based features may inadvertently learn associations between depression and gender that are not clinically relevant.

Case Study 1 demonstrates significant differences among demographic groups (i.e., gender, race/ethnicity) in terms of the ML labels (i.e., anxiety; Table 5). Case Study 2 further demonstrates differences in terms of the PHQ-8 scores and the ratio of patients with depression between female and male speakers, but this difference is not statistically significant. These differences have been found in prior psychological studies. For example, the higher global prevalence of depression among women compared to men is well-documented (65–67). In a study that presents a systematic review of the epidemiological literature, the global 12-month prevalence of major depressive disorder was 5.8% in females and 3.5% in males (67). Similarly, in two meta-analyses on gender differences in depression in nationally representative U.S. samples, the odds ratio was 1.95 for gender differences in diagnoses of major depression and the effect size for gender differences in depression symptoms was d=0.27 (68). This difference might be attributed to psychological factors (e.g., increased sensitivity to interpersonal relationships among women) and biological factors (e.g., hormonal changes throughout the lifespan) (64). Prior work further indicates that women report higher anxiety patterns compared to men (69, 70), which might be partially due to their nature and upbringing. This is consistent to results in Case Study 1, in which female speakers demonstrated higher anxiety scores than male speakers.

Despite these conceptually-grounded and potentially meaningful differences in the considered outcomes among demographic groups, the ML models were partially able to preserve those differences in their predictions. In Case Study 1, Hispanic male speakers depicted significantly higher levels of self-reported anxiety compared to White male speakers, but this difference between the two groups was not maintained in terms of estimated anxiety (Tables 3, 5). In addition, African American female and male speakers self-reported significantly lower anxiety than White female and male speakers, respectively, with this difference not being maintained in terms of estimated anxiety by the ML models (Tables 3, 5). In Case Study 2, the ML models that used the original acoustic features without any transformation underestimated depression for both female and male speakers (Table 8). When using the transformed features, the ML models overestimated depression for the male participants resulting in significantly higher ML-estimated PPR compared to base depression rate (Table 8, Figures 1E, F). Mixed findings were observed for female participants, where the ML models both overestimated and underestimated depression without yielding statistically significant differences between ML-estimated and base depression rate (Table 8, Figures 1E, F).

The ML models’ reduced ability to preserve significant differences among demographic groups in the outcomes can be attributed to several factors such as model design, data representation, and feature interactions. The models used (i.e., linear regression in Case Study 1, feedforward neural network with two hidden layers in Case Study 2) might be too simplistic or lack the capacity to capture subtle variations in the data, overlooking important differences between demographic groups. The nature of the data itself may further contribute to the difficulty in preserving significant differences. For Case Study 2, for example samples from the positive class were fewer compared to the ones from the negative class (e.g., depression samples were only 28.9% of the data). This can potentially be a reason why the ML models were not able learn robust representations that generalize well across different populations. As part of our future work, we will explore re-weighting methods that increase the importance of samples from the sensitive groups during training, thus might be able to reduce discrepancies between actual and estimated outcomes of these groups (20, 51, 53). Finally, the small sample size might have resulted in the ML models not being able to adequately capture the potentially complex and non-linear associations between features and demographic variables. Leveraging data augmentation methods, such as using contrastive learning to compare samples from underrepresented demographic groups with the rest of the population (71), could potentially mitigate this issue.

Overall, our results indicate that while the identification of variations in mental health labels across different demographics is well-documented in psychological studies, the ML models used in this paper only partially preserved the conceptually-grounded and potentially meaningful differences in their predictions. Designing and implementing a ML system involves many crucial decisions that can affect its effectiveness and efficiency and potentially lead to inaccuracies and misinterpretations in automated health assessments, particularly when dealing with conditions influenced by factors specific to gender or race/ethnicity. Findings of this paper raise awareness on the importance of thoughtful design of ML algorithms with a focus on preserving conceptually meaningful differences among demographic groups and practicing ongoing vigilance in the development and deployment of these ML systems.

In terms of differences in ML performance among groups, results for Case Study 1 were mixed. There were experimental configurations in which the sensitive groups, such as African Americans and Hispanics, depicted the lowest performance, but this was not always the case. A potential reason for that might be the small sample size of the sensitive groups. Results for Case Study 2 were a bit more clear and depression was more correctly identified for female speakers compared to male speakers. Prior work in emotion recognition has observed performance differences between female and male speakers (15, 16), but results as to which demographic group depicts highest ML accuracy were not conclusive. For instance, Sagha et al. showed that face-based emotion recognition was better for female speakers (16), while Gorrostieta et al. found that speech-based emotion recognition had better performance for male speakers (15).

Results from Case Study 2 indicate that there is no ML bias when using the original features, but these features yield accuracies close to chance, so they cannot be used in a meaningful manner for the purpose of depression classification. Depression classification accuracies improve above chance when we conduct feature selection followed by feature transformation, yet this introduces bias in favor of female and against male speakers. Certain configurations of the ML systems are able to mitigate this bias and result in improved depression classification accuracies (e.g., the K=10 most relevant features to depression followed by feature transformation yield BA of 65.44% and 62.96% for female and male speakers, respectively and EO=0.976). Bias mitigation is slightly more effective when we select the K most relevant features to depression followed by feature transformation, as opposed to removing the M features that are most relevant to gender followed by feature transformation. Nonetheless, it remains uncertain whether the above would translate effectively to similar types of speech datasets. Further exploration and testing across would be essential to validate the broader utility and effectiveness of these feature selection and transformation methods in mitigating bias.

The findings of this paper should be considered in the light of the following limitations. Data from both case studies had a small sample size (i.e., N=30 for Case Study 1; N=107 for Case Study 2). While it might be easier to obtain larger sample size for studies that examine non-continuous variables (e.g., electronic health records), it is not always easy to collect continuous speech and multimodal data from thousands of participants, since this usually requires the physical presence of participants in the lab. Online studies conducted via teleconferencing systems can potentially overcome this challenge and yield richer datasets. The majority of publicly available speech datasets on emotion and mental health contain limited meta-information, either in order to protect the participants’ identities or because the retention of detailed demographic information was not a common practice at the time when these datasets were developed. It would be important to seek information pertaining to detailed demographics as part of the future work and examine algorithmic bias at the intersection of age, gender, ethnicity, gender, and race. Finally, this study only investigated acoustic features from speech. Prior work demonstrates demographic leakage in human-centered ML models that are based on linguistic markers (62), thus it would be important to examine demographic bias in both linguistic and paralinguistic information.

## 6 Conclusion

We examined sources of algorithmic bias in speech-based models of health outcomes via two different studies, which allude to converging conclusions. Results from both studies indicate significant differences in terms of gender, race/ethnicity, and their intersection) for acoustic measures of frequency, spectral balance, and energy, which might be a reason contributing to the differential ML performance among the groups. Differences in ML performance were observed, but patterns were not consistent across all configurations. Finally, ML models do not preserve meaningful differences in estimated outcomes among groups; the considered health labels depicted significant differences across groups and while these differences are conceptually meaningful based on prior work, they were partially preserved in the ML decisions.

## Data Availability

The original contributions presented in the study are included in the article/Supplementary Material, further inquiries can be directed to the corresponding author.
