# Supplementary material for: Deconstructing demographic bias in speech-based machine learning models for digital health
Source: Front Digit Health. 2024 Jul 25;6:1351637. doi: 10.3389/fdgth.2024.1351637 (PMC11306200; doi:10.3389/fdgth.2024.1351637)
Supplement: Supplementary file 1 [file Datasheet1.pdf]

Supplementary Material

Table S1. The 20 most discriminative features of depression in decreasing order of Pearson's correlation with the PHQ label

Feature	Group	Pearson's correlation, p-value
F3bandwidth_sma3nz_amean *	Frequency	$r = -0.071, p = 0$
F0semitoneFrom27.5Hz_sma3nz_percentile50.0 *	Frequency	$r = 0.062, p = 0$
F0semitoneFrom27.5Hz_sma3nz_percentile20.0 *	Frequency	$r = 0.061, p = 0$
F0semitoneFrom27.5Hz_sma3nz_amean *	Frequency	$r = 0.061, p = 0$
F0semitoneFrom27.5Hz_sma3nz_percentile80.0 *	Frequency	$r = 0.061, p = 0$
logRelF0-H1-H2_sma3nz_amean	Spectral balance	$r = 0.058, p = 0$
equivalentSoundLevel_dBp *	Energy/amplitude	$r = -0.054, p = 0$
mfcc2V_sma3nz_amean *	Spectral balance	$r = 0.053, p = 0$
HNRdBACF_sma3nz_amean *	Energy/amplitude	$r = 0.053, p = 0$
F1amplitudeLogRelF0_sma3nz_amean	Spectral balance	$r = -0.052, p = 0$
mfcc2_sma3_amean	Energy/amplitude	$r = 0.052, p = 0$
hammarbergIndexV_sma3nz_amean *	Spectral balance	$r = 0.052, p = 0$
F3amplitudeLogRelF0_sma3nz_amean	Spectral balance	$r = -0.051, p = 0$
F2amplitudeLogRelF0_sma3nz_amean	Spectral balance	$r = -0.051, p = 0$
logRelF0-H1-A3_sma3nz_amean *	Spectral balance	$r = 0.051, p = 0$
loudnessPeaksPerSec	Energy/amplitude	$r = 0.051, p = 0$
F1amplitudeLogRelF0_sma3nz_stddevNorm	Spectral balance	$r = 0.048, p = 0$
F2bandwidth_sma3nz_amean *	Frequency	$r = -0.048, p = 0$
F3amplitudeLogRelF0_sma3nz_stddevNorm	Spectral balance	$r = 0.048, p = 0$
MeanUnvoicedSegmentLength	temporal	$r = 0.048, p = 0$

$*$: Feature with statistically significant difference ($p < 0.01$) between female and male speakers.

Table S2. The 20 most discriminative features of biological sex, depicted in increasing order of the $p - value$ of the t-test comparing between female and male speakers

Feature	Group	T-statistic, p-value
F1Frequency_sma3nz_amean	Frequency	$t = 1.424, p = 0.157$
mfcc4_sma3_stddevNorm	Spectral balance	$t = -1.420, p = 0.158$
F0semitoneFrom27.5Hz_sma3nz_meanFallingSlope	Frequency	$t = -1.372, p = 0.172$
equivalentSoundLevel_dBp	Energy/amplitude	$t = 1.371, p = 0.173$
F2Frequency_sma3nz_amean	Frequency	$t = 1.359, p = 0.176$
F3Frequency_sma3nz_amean	Frequency	$t = 1.343, p = 0.181$
logRelF0-H1-H2_sma3nz_stddevNorm	Spectral balance	$t = -1.333, p = 0.185$
F0semitoneFrom27.5Hz_sma3nz_percentile80.0	Frequency	$t = -1.302, p = 0.195$
F0semitoneFrom27.5Hz_sma3nz_pctlrange0-2	Frequency	$t = -1.279, p = 0.203$
F1amplitudeLogRelF0_sma3nz_amean	Spectral balance	$t = 1.239, p = 0.218$
F3amplitudeLogRelF0_sma3nz_amean	Spectral balance	$t = 1.228, p = 0.222$
F2amplitudeLogRelF0_sma3nz_amean	Spectral balance	$t = 1.221, p = 0.224$
F0semitoneFrom27.5Hz_sma3nz_percentile50.0	Frequency	$t = -1.218, p = 0.225$
mfcc1_sma3_amean	Energy/amplitude	$t = -1.181, p = 0.239$
hammarbergIndexUV_sma3nz_amean	Spectral balance	$t = -1.140, p = 0.256$
F0semitoneFrom27.5Hz_sma3nz_amean	Frequency	$t = -1.139, p = 0.257$
mfcc1V_sma3nz_amean	Energy/amplitude	$t = -1.090, p = 0.277$
loudnessPeaksPerSec	Energy/amplitude	$t = -1.086, p = 0.279$
jitterLocal_sma3nz_stddevNorm	Frequency	$t = -1.034, p = 0.303$
mfcc4V_sma3nz_stddevNorm	Energy/amplitude	$t = -1.033, p = 0.303$
